# Smoking as a Risk Factor for Surgical Site Complications in Implant-Based Breast Surgery

**DOI:** 10.7759/cureus.18876

**Published:** 2021-10-18

**Authors:** Isaac Zucker, Antoun Bouz, Grettel Castro, Pura Rodriguez de la Vega, Noel C Barengo

**Affiliations:** 1 Department of Translational Medicine, Florida International University, Herbert Wertheim College of Medicine, Miami, USA; 2 Department of Public Health, University of Helsinki, Helsinki, FIN; 3 Faculty of Medicine, Riga Stradins University, Riga, LVA

**Keywords:** wound breakdown, wound dehiscence, surgical infections, implant-based breast augmentation, smoking tobacco

## Abstract

Background

Smoking is a cause of many postoperative complications, including delayed wound healing, tissue necrosis, and reconstructive flap loss. However, there is a paucity of evidence-based guidelines for smoking cessation in patients undergoing implant-based breast surgery.

Objective

The objective of this study was to determine if smoking is associated with wound dehiscence or superficial/deep surgical site infection (SSI) in women undergoing implant-based breast surgery.

Methods

Using theAmerican College of Surgeons National Surgical Quality Improvement Program, data was obtained of U.S. adult females (n=10,077) between the ages of 18 and 70 who underwent insertion of a breast prosthesis from 2014 to 2016. The patient’s preoperative smoking status, demographics, and comorbidities were analyzed to determine association with wound dehiscence, superficial SSI, and deep SSI. Unadjusted and adjusted logistic regression analyses were used to calculate odds ratios (OR) and 95% confidence intervals (95% CI).

Results

Patients who smoked had a statistically significant higher proportion of wound complications (2.4%) compared to non-smokers (1.3%; p<0.01). Adjusted analysis demonstrated a significantly higher odds of wound complications in smoking patients compared to those who did not smoke (OR 2.0; 95% CI 1.3-3.2).

Conclusions

Our study suggests that smoking is an independent risk factor for postoperative complications in patients undergoing implant-based breast surgery. These results have significant clinical implications, as increased precautions can be taken in smokers undergoing breast surgery to minimize postoperative wound complications. Future studies may determine the optimal amount of time that patients should abstain from smoking prior to implant-based breast surgery.

## Introduction

Smoking rates in the U.S. have dropped substantially from 20.9% in 2005 to approximately 13.7% in 2018, with nearly 12% of all women being current smokers. Although this is a significant drop, it still translates to an estimated 34.2 million adults in the United States that continue to smoke cigarettes [1]. An increasing number of implant-based breast surgeries are being performed by plastic surgeons in the United States annually. Implant-based breast augmentation is the most popular cosmetic procedure in the United States, with over 314,000 surgeries completed in 2018 [2]. Additionally, implant-based breast reconstruction is the most commonly performed type of reconstructive breast surgery performed in the United States, largely outnumbering autologous techniques [3].

Previous literature has demonstrated that cigarette smoke delays wound healing in most surgical procedures [4,5], including neurological surgery [6], otologic surgery [7], and various types of plastic and reconstructive surgeries, in which postoperative surgical site complications are already common. Smoking increases the rates of wound complications in nipple reconstruction, autologous breast reconstruction, facelifts, flap-based reconstruction, and implant-based reconstruction following oncologic procedures [8-10].

Although several studies have already described the role of smoking in delaying wound healing in breast reconstructive procedures following oncologic therapy, there is little literature addressing the impact of preoperative smoking in the general population of patients receiving breast implants [8,10-13]. Current evidence-based guidelines suggest that women undergoing postmastectomy expander/implant breast reconstruction should be counseled regarding smoking cessation. However, such recommendations for women undergoing breast augmentation are sparce [14].

Thus, the objective of this study was to determine if smoking is associated with wound dehiscence or superficial/deep surgical site infection (SSI) in women undergoing implant-based breast surgery.

## Materials and methods

Study design

The study design was a non-concurrent cohort study analyzing secondary data using information retrieved from the 2014 to 2016 American College of Surgeons National Surgical Quality Improvement Program databases (ACS NSQIP).

Study population

The 2014 to ACS NSQIP database was accessed on May 13, 2020. Utilizing direct chart review, the ACS NSQIP database collects information from patients undergoing surgical procedures in both outpatient and inpatient settings. The ACS NSQIP obtains data from patients in the preoperative, intraoperative, and up to 30 days postoperative setting. Patients who had undergone plastic surgery procedures of interest were identified using current procedural terminology (CPT) codes. The major inclusion criteria were as follows: women between the ages of 18-70 who have undergone surgery for insertion of prosthesis following a mastopexy, mastectomy, or reconstruction or had a prosthetic implant placed. Participants were excluded from analysis if they were missing data from ACS NSQIP regarding the independent variables, dependent variables, and co-variants for the study. Participants were also excluded from the study if they had undergone autologous flap-based breast reconstruction without the placement of a prosthesis. The final sample size was 10,077 patients.

Measures

Smoking status was obtained from the ACS NSQIP database. Patients who smoked within one year of their surgery were considered smokers, and those who did not smoke within one year of their surgery were considered non-smokers. Smoking history beyond one year was not available, and thus past smoking status could not be established. Smoking status was a self-reported outcome, and recorded as “Yes” for current smokers and “No” for non-smokers.

Wound complications were defined as one or more of the following three distinct outcome variables: wound dehiscence, superficial SSI, and/or deep SSI (Table 1). They were obtained from the outcomes reported in the ACS NSQIP. Wound dehiscence was reported if there was a total failure/breakdown of the surgical closure, which compromised the integrity of the procedure. Superficial SSI was defined as an infection occurring within 30 days postoperatively involving only the skin or subcutaneous tissue of the incision site with at least one of the following: purulent drainage from the incision, with or without laboratory confirmation; isolation of organisms from a culture of fluid or tissue from the incision; fever, pain or tenderness, localized swelling, redness, or heat and the incision is deliberately opened by the surgeon; or diagnosis of superficial site infection by the surgeon or attending physician. Stitch abscesses, infected burn wounds, and incisional SSI that extends into the fascia and muscle were not reported as superficial SSI. Deep SSI was defined as an infection occurring within 30 days postoperatively involving deep soft tissues (fascia and muscle) of the incision site and including at least one of the following: purulent drainage from the incision but not the organ/space component of the surgical site; a deep incision that spontaneously dehisces or is reopened by the surgeon when the patient has at least one of the cardinal symptoms of infection (fever >38°C, localized pain or tenderness, unless culture is negative); an abscess or other evidence of infection involving the incision (noted on physical exam, reoperation, or via imaging studies); or diagnosis of a deep SSI by a surgeon or attending physician [15].

**Table 1:** Description of wound complications as defined by the ACS NSQIP. SSI: surgical site infection. ACS NSQIP: American College of Surgeons National Surgical Quality Improvement Program.

Wound infection	Definition	Additional variables
Superficial wound infection	Infection occurring within 30 days postoperatively involving only the skin or subcutaneous tissue of the incision site	Must include one or more of the following: (1) Purulent drainage from the incision, with or without laboratory confirmation. (2) Isolation of organisms from a culture of fluid or tissue from the incision. (3) Any sign or symptom of infection: fever, pain or tenderness, localized swelling, redness, or heat and the incision is deliberately opened by the surgeon. (4) Diagnosis of superficial site infection by the surgeon or attending physician
Deep wound infection	Infection occurring within 30 days postoperatively involving deep soft tissues (fascia and muscle) of the incision site	Must include one or more of the following: (1) Purulent drainage from the incision but not the organ/space component of the surgical site. (2) A deep incision that spontaneously dehisces or is reopened by the surgeon. (3) Any sign or symptom of infection: fever >38°C, localized pain or tenderness, unless culture is negative. (4) Abscess or other evidence of infection involving the incision (noted on physical exam, reoperation, or via imaging studies. (5) Diagnosis of a deep SSI by a surgeon or attending physician
Wound dehiscence	Completed failure or breakdown of surgical closure	

Other covariates included in the study were age, sex, race/ethnicity, BMI, diabetes mellitus status, dyspnea, functional health status, steroid/immunosuppressant use, >10% loss of body weight in six months prior to surgery, and bleeding disorders. The patient’s BMI measurement reflected the most recently documented weight/height reported in their medical records. Diabetes status was divided into diabetic (requiring insulin), diabetic (not requiring insulin), and non-diabetic. Hemoglobin A1 (HbA1) and blood glucose are not reported in the NSQIP, so disease control could not be inferred. Functional health status is reflective of a patient’s activities of daily living (ADLs: bathing, feeding, dressing, toileting, and mobility). The value reported was the best functional health status obtained from the patient in the 30 days prior to surgery and is reported as: independent; partially dependent; totally dependent; unknown. Dyspnea, steroid status, bleeding disorders, weight loss, and steroid/immunosuppressant use were obtained from their most recent medical report.

Statistical analysis

Data analysis was performed using Stata/MP version 15.2 (StataCorp, College Station, TX). Baseline characteristics and comorbidities were reported as raw counts and percentages for nominal variables. A bivariate chi-squared analysis was utilized in order to identify possible confounding variables. Next, a collinearity diagnostic assessment was performed to determine if variables were correlated with one another. The correlation between smoking status and wound complications was assessed using a Pearson correlation test. Finally, unadjusted and adjusted logistic regression models were performed to calculate odds ratios (OR) and their respective 95% confidence intervals (95% CI).

## Results

Table 2 compares the characteristics of patients who underwent breast reconstruction found in the NSQIP database from 2014 to 2016 by smoking status. There was a higher proportion of white participants in the smoking group (89.3%) compared to the non-smoking group (87.1%, p=0.08). Additionally, participants in the non-smoking group had a higher mean age (46.2 years) compared to those in the smoking group (43.7 years, p<0.001). Dyspnea was more frequently seen in smokers (1.6%) compared to non-smokers (0.9%, p<0.05). There was no statistically significant difference found in the distribution of race (p=0.08), BMI (p=0.85), diabetes status (p=0.07), functional health status (p=0.32), steroid use (p=0.33), and history of bleeding disorder (p=0.53) between those who were smokers and those who were not.

The unadjusted and adjusted associations between the characteristics of patients undergoing breast implants and wound complications are presented in Table 3. Before adjusting for covariates patients who smoked within the last year had an odds ratio for wound complications of 1.9 [95% confidence interval (CI) 1.2-2.9] when compared with patients who did not smoke. Age was not associated with an increased risk for wound complications (OR 1.0; 95% CI 1.0-1.0). The corresponding increase in odds for those with a BMI >30 kg/m^2^ was 130% when compared with BMI <30 kg/m^2^ (OR 2.3; 95 CI 1.6-3.3). Finally, having diabetes was not associated with a statistically significant increased likelihood to develop wound complications compared with patients who did have diabetes (OR 1.8; 95% CI 0.8-3.5).

After adjusting for covariates, patients who smoked within the last year had a statistically significant increase in wound complications compared with patients who did not smoke (OR 2.0; 95% CI 1.3-3.2). However, age was not associated with wound complications (OR 1.0; 95% CI:1.0-1.0). The patients who had a BMI >30 kg/m^2^ had a twofold increase in odds for developing wound complications (95% CI: 1.4-2.9). Finally, diabetes was not associated with wound complication rate compared with non-diabetics (OR 1.1; 95% CI 0.6-2.3).

**Table 2:** Baseline characteristics for smokers and non-smokers undergoing implant-based breast surgery during 2014, 2013, and 2016.

Patient characteristics	Non-smoker	Smoker	P-value
	N (%)	N (%)	
Age (years)–mean (SD)	46.2 (12.7)	43.7 (12.2)	<0.001
Race			0.08
White	6698 (87.1)	764 (89.3)	
Non-White	989 (12.9)	92 (10.8)	
BMI			0.85
BMI <30 kg/m^2^	7466 (82.9)	839 (83.2)	
BMI >30 kg/m^2^	1339 (17.1)	170 (16.9)	
Diabetes status			0.07
No	8720 (96.2)	983 (97.3)	
Yes	347 (3.8)	27 (2.7)	
Dyspnea			0.04
No	8985 (99.1)	994 (98.4)	
Yes	82 (0.9)	16 (1.6)	
Functional health status			0.32
Independent	8978 (99.5)	1002 (99.7)	
Partially dependent	48 (0.5)	3 (0.3)	
Steroid use for chronic condition			0.33
No	8954 (98.8)	1001 (99.1)	
Yes	113 (1.3)	9 (0.9)	
Bleeding condition			0.53
No	9007 (99.3)	1005 (99.5)	
Yes	60 (0.7)	5 (0.5)	

**Table 3:** Unadjusted and adjusted odds ratio for postoperative wound complications undergoing implant-based breast surgery during 2014, 2013, and 2016. OR: odds ratio; CI: confidence interval; Ref: reference group.

Patient characteristics	Unadjusted OR (95% CI)	Adjusted OR (95% CI)
Smoking status		
No	Ref	Ref
Yes	1.9 (1.2-2.9)	2.0 (1.3-3.2)
Age	1.0 (1.0-1.0)	1.0 (1.0-1.0)
BMI		
BMI <30 kg/m^2^	Ref	Ref
BMI >30 kg/m^2^	2.3 (1.6-3.3)	2.0 (1.4-2.9)
Diabetes mellitus		
No	Ref	Ref
Yes	1.8 (0.9-3.5)	1.1 (0.6-2.3)

## Discussion

Our data revealed that smoking is an independent risk factor for the development of wound dehiscence, superficial SSI, or deep SSI in women receiving breast implants. Obese patients were also more likely to experience postoperative wound complications than non-obese patients. However, diabetes was not associated with postoperative wound complications.

These results are consistent with the findings in the current scientific literature showing that smoking has a negative impact on wound healing in many populations [4-8,10-11,16-19]. Previous literature has established smoking as a risk factor for postoperative surgical complications. Fischer et al. and Pechevy et al. demonstrated that smoking was an independent risk factor for wound dehiscence in women undergoing breast reconstruction [11,12]. Joy et al. strengthened these findings with their study, which suggested increased rates of wound dehiscence in smoking patients undergoing breast oncological and reconstructive surgery [8]. Obese smokers were four times as likely to develop postoperative wound dehiscence compared to non-smokers [10]. Finally, multiple studies established the association between smoking and increased rates of postoperative wound infection and surgical complications in women undergoing breast reconstruction surgery [16,17,20]. Our study corroborates and expands upon the general consensus of the existing literature. Previous studies focused on the impact of perioperative smoking in patients undergoing reconstructive surgery after oncological procedures, while our current study includes patients who received breast implants for any reason. This expands our target population to include patients undergoing cosmetic breast augmentation. Contrary to previous findings, our study did not find an association between diabetes status, a well-described predictor of poor wound healing, and increased rates of wound complications [11,21-23].

The mechanism by which cigarette smoke impacts wound healing is complex and multifaceted [5,24-26]. Previous studies describe the deleterious effects of smoking on wound healing by focusing on the components of cigarettes: nicotine, carbon monoxide, and hydrogen cyanide. Nicotine is a sympathomimetic agent, triggering the release of norepinephrine which ultimately results in peripheral vasoconstriction that leads to tissue hypoperfusion [5,25,26]. Nicotine also enhances platelet adhesion, resulting in the formation of micro-clots that further decrease perfusion [19]. Carbon monoxide is also a major contributor to decreased tissue oxygenation, as it binds to hemoglobin with a much higher affinity than oxygen [24]. Cyanide inhibits major enzymes involved in oxidative metabolism, compromising oxygen consumption in tissue that play a pivotal role in wound healing processes [19,24,26]. Previous literature suggests that cigarette smoke increases myofibroblast proliferation due to increased fibrinogen and fibronectin, which leads to increased postoperative wound contraction [26]. Finally, cigarette smoke is thought to impair fibroblast function, thus reducing neovascularization and wound remodeling [19,26]. Overall, smoking impairs the natural wound healing process by decreasing oxygen availability and breakdown, while inducing unfavorable molecular changes through alterations of fibroblast and myofibroblast function.

Our study’s secondary findings are also consistent with those of previous studies. Obese patients had significantly higher rates of wound dehiscence, superficial SSI, and deep SSI compared to non-obese patients. Previous studies have suggested that obesity is a significant risk factor for wound complications in surgical patients [10,18,24]. Some studies have postulated that the wounds in obese patients are under increased tension, which leads to decreased perfusion and thus a decreased availability of oxygen that is required for normal wound repair processes [27]. Decreased tissue perfusion may translate into decreased delivery of antibiotics to surgical sites, increasing the likelihood of wound infection [24], and increased wound tension may increase the likelihood of wound dehiscence [27]. Interestingly, our study results suggest that age acts as a negative confounder to wound breakdown in patients undergoing implant-based breast surgery. When we controlled for patient age, the odds of wound complications in smokers actually increased. Previous literature suggests that older patients heal more slowly than younger patients and are at increased risk for postoperative wound complications [24,28,29].

Our study is not without limitations. First, the ACS-NSQIP reports patients as current smokers if they have smoked within one year. However, more detail regarding patients’ smoking status may be clinically revealing and allow us to draw conclusions regarding appropriate recommendations for perioperative smoking cessation. Second, the ACS-NSQIP only reports 30-day outcomes, and thus the incidence of long-term complications may be underreported. In addition, additional variables such as reject of the prosthesis or skin necrosis as well as chronic disorders were not captured in the database. Unfortunately, the ACS-NSQIP fails to report adjuvant or neoadjuvant chemoradiation therapy, which are well-known causes of impaired wound healing in patients who have undergone oncological therapy, and thus we could not include those variables in our multivariate analysis. Overall, we were able to capture a large sample of patients who underwent breast implant placement and are confident that our study results can be applied to future patients undergoing implant-based breast surgery.

## Conclusions

In conclusion, our study suggests that smoking is an independent risk factor for postoperative complications in patients undergoing implant-based breast surgery. These results have significant clinical implications, as increased precautions can be taken in smokers undergoing breast surgery in order to minimize post-operative wound complications. Additionally, this population of patients may experience improved outcomes if perioperative smoking cessation is discussed prior to surgery. Although our study was able to establish a significant relationship between smoking and postoperative wound complications in these patients, further studies need to be conducted in order to determine the optimal amount of time that patients should abstain from smoking prior to implant-based breast surgery.

## References

[1] National Center for Chronic Disease Prevention and Health Promotion (US) Office on Smoking and Health (2014). The Health Consequences of Smoking—50 Years of Progress: A Report of the Surgeon General. Prevention.

[2] (2020). American Society of Plastic Surgeons. National clearinghouse of plastic surgery procedural statistics, 2019. https://www.plasticsurgery.org/documents/News/Statistics/2019/plastic-surgery-statistics-full-report-2019.pdf.

[3] Albornoz CR, Bach PB, Mehrara BJ (2013). A paradigm shift in U.S. breast reconstruction: increasing implant rates. Plast Reconstr Surg.

[4] Khullar D, Maa J (2012). The impact of smoking on surgical outcomes. J Am Coll Surg.

[5] Sørensen LT (2012). Wound healing and infection in surgery. The clinical impact of smoking and smoking cessation: a systematic review and meta-analysis. Arch Surg.

[6] Lau D, Berger MS, Khullar D, Maa J (2013). The impact of smoking on neurosurgical outcomes. J Neurosurg.

[7] Kay-Rivest E, Mascarella M, Sewitch MJ, Cloutier F, Mijovic T (2020). Association between smoking and 30-day outcomes in otologic surgery. Otolaryngol Head Neck Surg.

[8] Joy MT, Rich MD, Moyer KE (2018). Axillary lymphadenectomy and wound complications in implant-based breast reconstruction. Ann Plast Surg.

[9] Knobloch K, Gohritz A, Reuss E, Vogt PM (2008). Nicotine in plastic surgery: a review. Chirurg.

[10] Rudolph M, Moore C, Pestana IA (2019). Operative risk stratification in the obese female undergoing implant-based breast reconstruction. Breast J.

[11] Fischer JP, Nelson JA, Au A, Tuggle CT III, Serletti JM, Wu LC (2014). Complications and morbidity following breast reconstruction--a review of 16,063 cases from the 2005-2010 NSQIP datasets. J Plast Surg Hand Surg.

[12] Pechevy L, Carloni R, Guerid S, Vincent PL, Toussoun G, Delay E (2017). Skin-reducing mastectomy in immediate reconstruction: how to limit complications and failures. Aesthetic Surg J.

[13] Klasson S, Nyman J, Svensson H, Velander P (2016). Smoking increases donor site complications in breast reconstruction with DIEP flap. J Plast Surg Hand Surg.

[14] Alderman A, Gutowski K, Ahuja A, Gray D (2014). ASPS clinical practice guideline summary on breast reconstruction with expanders and implants. Plast Reconstr Surg.

[15] American College of Surgeons, N N (2020). American College of Surgeons, NSQIP, user guide for the 2012 ACS NSQIP participant use data file. https://www.facs.org/~/media/files/quality%20programs/nsqip/ug12.ash.

[16] Goodwin SJ, McCarthy CM, Pusic AL (2005). Complications in smokers after postmastectomy tissue expander/implant breast reconstruction. Ann Plast Surg.

[17] Lardi AM, Ho-Asjoe M, Mohanna PN, Farhadi J (2014). Immediate breast reconstruction with acellular dermal matrix: factors affecting outcome. J Plast Reconstr Aesthetic Surg.

[18] Olsen MA, Lefta M, Dietz JR (2008). Risk factors for surgical site infection after major breast operation. J Am Coll Surg.

[19] Silverstein P (1992). Smoking and wound healing. Am J Med.

[20] Hill JL, Wong L, Kemper P, Buseman J, Davenport DL, Vasconez HC (2012). Infectious complications associated with the use of acellular dermal matrix in implant-based bilateral breast reconstruction. Ann Plast Surg.

[21] Okonkwo UA, DiPietro LA (2017). Diabetes and wound angiogenesis. Int J Mol Sci.

[22] Hart A, Funderburk CD, Chu CK (2017). The impact of diabetes mellitus on wound healing in breast reconstruction. Ann Plast Surg.

[23] Baltzis D, Eleftheriadou I, Veves A (2014). Pathogenesis and treatment of impaired wound healing in diabetes mellitus: new insights. Adv Ther.

[24] Guo S, Dipietro LA (2010). Factors affecting wound healing. J Dent Res.

[25] Ahn C, Mulligan P, Salcido RS (2008). Smoking-the bane of wound healing: biomedical interventions and social influences. Adv Skin Wound Care.

[26] Sørensen LT (2012). Wound healing and infection in surgery: the pathophysiological impact of smoking, smoking cessation, and nicotine replacement therapy: a systematic review. Ann Surg.

[27] Wilson JA, Clark JJ (2004). Obesity: impediment to postsurgical wound healing. Adv Skin Wound Care.

[28] Gerstein AD, Phillips TJ, Rogers GS, Gilchrest BA (1993). Wound healing and aging. Dermatol Clin.

[29] Jones PL, Millman A (1990). Wound healing and the aged patient. Nurs Clin North Am.

